# Survival-related genes are diversified across cancers but generally enriched in cancer hallmark pathways

**DOI:** 10.1186/s12864-022-08581-x

**Published:** 2022-05-04

**Authors:** Po-Wen Wang, Yi-Hsun Su, Po-Hao Chou, Ming-Yueh Huang, Ting-Wen Chen

**Affiliations:** 1 Institute of Bioinformatics and Systems Biology, National Yang Ming Chiao Tung University, Hsinchu, 30068 Taiwan; 2 Center for Intelligent Drug Systems and Smart Bio-devices (IDS2B), National Yang Ming Chiao Tung University, Hsinchu, 30068 Taiwan; 3 Industrial Development PhD Program of the College of Biological Science and Technology, National Yang Ming Chiao Tung University, Hsinchu, 30068 Taiwan; 4 Institute of Statistical Science, Academia Sinica, Taipei, 11529 Taiwan; 5 Department of Biological Science and Technology, National Yang Ming Chiao Tung University, Hsinchu, 30068 Taiwan

**Keywords:** Survival, Pan-cancer, Biomarker, Univariate analysis, Cox proportional hazards model, Log-rank test

## Abstract

**Background:**

Pan-cancer studies have disclosed many commonalities and differences in mutations, copy number variations, and gene expression alterations among cancers. Some of these features are significantly associated with clinical outcomes, and many prognosis-predictive biomarkers or biosignatures have been proposed for specific cancer types. Here, we systematically explored the biological functions and the distribution of survival-related genes (SRGs) across cancers.

**Results:**

We carried out two different statistical survival models on the mRNA expression profiles in 33 cancer types from TCGA. We identified SRGs in each cancer type based on the Cox proportional hazards model and the log-rank test. We found a large difference in the number of SRGs among different cancer types, and most of the identified SRGs were specific to a particular cancer type. While these SRGs were unique to each cancer type, they were found mostly enriched in cancer hallmark pathways, e.g., cell proliferation, cell differentiation, DNA metabolism, and RNA metabolism. We also analyzed the association between cancer driver genes and SRGs and did not find significant over-representation amongst most cancers.

**Conclusions:**

In summary, our work identified all the SRGs for 33 cancer types from TCGA. In addition, the pan-cancer analysis revealed the similarities and the differences in the biological functions of SRGs across cancers. Given the potential of SRGs in clinical utility, our results can serve as a resource for basic research and biotech applications.

**Supplementary Information:**

The online version contains supplementary material available at 10.1186/s12864-022-08581-x.

## Background

Many molecular features that can predict the clinical outcomes in cancers have been disclosed from large-scale cancer genome projects, such as The Cancer Genome Atlas (TCGA, https://www.cancer.gov/tcga), The International Cancer Genome Consortium (ICGC) and Therapeutically Applicable Research to Generate Effective Treatments (TARGET, https://ocg.cancer.gov/programs/target) [1, 2]. The predictive features could be the biological molecule itself or alterations/modifications of the biological molecule. For example, hypermethylation of *BRCA1* promoter is a predictor for the overall survival (OS) and the disease-free survival (DFS) in triple-negative breast cancer [3]. Signatures consisting of multiple hypermethylated or hypomethylated sites can stratify cancer patients into high-risk and low-risk groups which have significantly different OS outcomes for bladder urothelial carcinoma (BLCA), breast invasive carcinoma (BRCA), head and neck squamous cell carcinoma (HNSC), liver hepatocellular carcinoma (LIHC), lung adenocarcinoma (LUAD), thyroid carcinoma (THCA) and uterine corpus endometrial carcinoma (UCEC) [4]. Copy number variations (CNVs) show prognostic power in breast, endometrial, renal clear cell thyroid, colon-rectal and oral squamous cell carcinomas [5, 6]. In addition to the changes at the DNA level, changes at the expression levels of mRNA, lncRNA, miRNA, and protein are also potential biomarkers for predicting OS and DFS in cancers [7–9]. All the epigenetic variations, CNVs, and transcriptome alterations can result in the modifications of the proteome, and consequently influence the clinical outcome and prognosis.

Even though proteins are the direct players in regulating cancer-related pathways, comprehensive quantification of the proteome, which usually is performed by mass-spectrometry, is technically challenging [10]. Transcriptome quantification data derived from RNA-seq, on the other hand, are more popular for prognostic biomarker screening due to the rapid development of Next Generation Sequencing (NGS) technology. Benefiting from numerous publicly available expression profiles for cancers, databases are built for discovering the prognosis power of mRNA, miRNA, or lncRNA [11, 12]. Many studies have observed that the RNA expression levels are prognosis-related in individual cancers [13–16]. Once the prognostic genes were identified, the biological functions involved in these genes can be valuable in predicting treatment outcomes and hence may affect treatment decisions.

Making use of results in cancer genome projects, pan-cancer analyses have revealed the molecular distances among different cancer types and suggested a new classification of cancer types based on their aneuploidy, CpG hypermethylation, mRNA, lncRNA, miRNA, or protein [17–22]. These pan-cancer studies have disclosed the similarities among different type of cancers. For instance, based on the mRNA expression profiles, neural-related cancers, such as glioblastoma multiforme (GBM), brain lower-grade glioma (LGG), and pheochromocytoma and paraganglioma (PCPG), were grouped. Cancers originated from kidneys, such as kidney renal clear cell carcinoma (KIRC) and kidney renal papillary cell carcinoma (KIRP) but not kidney chromophobe (KICH), were clustered together [17]. Similarities and variations among different types of cancers may provide hints for the underlying biological mechanism of cancer developments, which could eventually lead to different clinical outcomes. To date, the majority of studies focused on identifying a combination of genes that can predict survival outcome, and single survival-related genes (SRGs) were usually ignored. However, their potentially prognostic powers remain relevant and may play roles in the cancer driver pathways on the molecular level. Hence, it is worthwhile to explore the SRGs at both the pan-cancer and the single cancer levels.

The log-rank test is a hypothesis test for comparing the survival distributions of two samples. It is non-parametric and so appropriate for sparse, skewed data of an unknown distribution, such as the data to which we applied it, namely a low expression group sample and a high expression group sample, to identify cancer SRGs. Cox regression, also known as proportional hazards regression, is a method for investigating the effect of several variables upon the time it takes a specified event to happen. It is semi-parametric, in that it does not assume a particular form for the underlying distribution, but it does depend on several technical assumptions, in particular that the unique effect of a unit increase in any given covariate is multiplicative with respect to the hazard rate. Both methods are widely used in clinical trials. The advantages of Cox regression is that it provides a numerical estimate of the difference between two groups, unlike the log rank test which merely flags whether a difference is significant or not at the specified level.

In this study, we systematically carried out a pan-cancer analysis of the SRGs involved in 33 cancers using data from the TCGA database. We applied both the log-rank test and Cox regression for the identification of SRGs. We identified all the genes whose expression levels were significantly correlated with clinical survival, for each cancer type. We further investigated the distribution of these genes across cancers and explored the pathways of these SRGs involved in different cancer types.

## Results

### Identification of SRGs in cancers using two statistical models

**Fig. 1:**
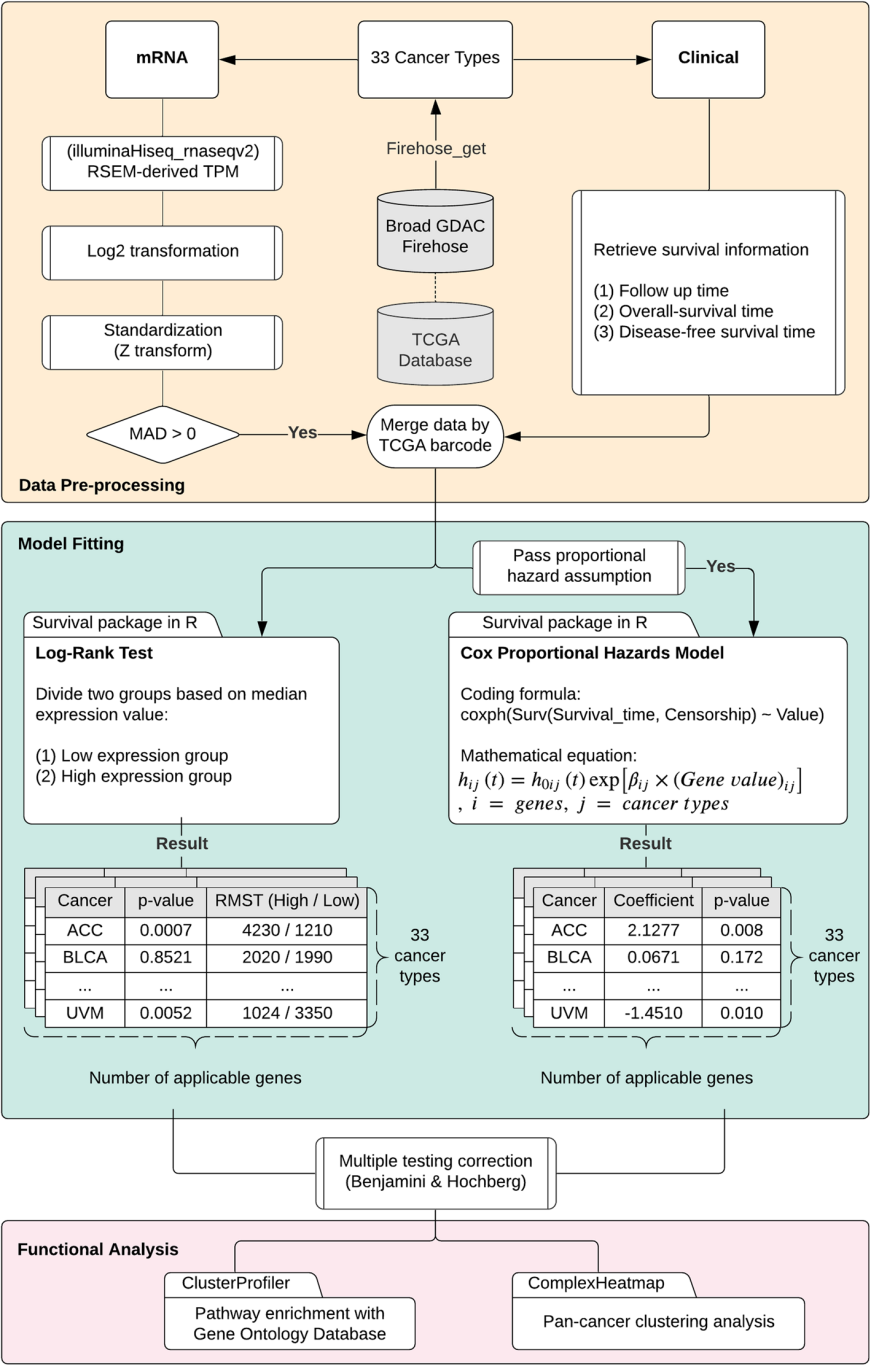
The workflow for data pre-processing, model fitting and functional analysis. The flowchart illustrates the working process of the present paper. RNA-Seq and clinical survival data were retrieved from Broad GDAC firehose. mRNA expression data from Illumina Hiseq were used. The RSEM-derived TPM were log2 transformed and standardized. Genes with median absolute deviation (MAD) greater than zero were fused with clinical survival data. In the model fitting section, the derived data were directly applied to the log-rank test or were examined for the proportional hazards assumption before applying the Cox model. Both models were fitted individually for each gene in each cancer type. The result tables indicate the simplified information generating from the models. Multiple testing corrections were performed before subsequently analysed by pathway enrichment and clustering. Abbreviation: RSEM, RNA-Seq by expectation maximization. RMST, restricted mean survival time. TPM, transcript per million

To identify SRGs, we used mRNA expression values and clinical survival times from the TCGA database. We selected 9133 patients with primary solid tumors and primary blood-derived tumors from 33 cancer types (Table 1). Two statistical methods, the log-rank test and the Cox proportional hazards model, were used in this study. An advantage of the log-rank test is that it relies on relatively few assumptions, but a disadvantage is that it cannot distinguish the extent of risks among predictors [23]. However, Cox regression estimates the change in the log hazard ratio for each one-unit increase in predictors. As shown in Fig. 1, genes were first screened by median absolute deviation (MAD) because we reasoned that only relatedly expressed genes are potentially associated with survival time. Furthermore, genes that violated the Cox proportional hazards assumption, i.e. a constant hazard ratio, were removed from the Cox analysis [24]. The number of the applicable genes for the two statistical models are listed in Table 1.

**Table 1:** Summary of 33 cancer types in TCGA

Cancer Type (Abbreviation)	Sample Number	Event Number	Applicable Genes	
			Log-Rank Test	Cox Regression
Adrenocortical carcinoma (ACC)	79	43	16,921	16,146
Bladder urothelial carcinoma (BLCA)	407	229	17,469	16,137
Breast invasive carcinoma (BRCA)	1080	204	17,658	12,023
Cervical squamous cell carcinoma and endocervical adenocarcinoma (CESC)	291	88	17,531	16,171
Cholangiocarcinoma (CHOL)	36	22	17,510	15,992
Colon adenocarcinoma (COAD)	279	105	17,454	16,698
Lymphoid Neoplasm Diffuse Large B-cell Lymphoma (DLBC)	47	16	16,897	16,439
Esophageal carcinoma (ESCA)	184	113	18,118	17,285
Glioblastoma multiforme (GBM)	152	131	17,655	16,469
Head and neck squamous cell carcinoma (HNSC)	519	271	17,699	16,248
Kidney chromophobe (KICH)	65	12	17,175	16,421
Kidney renal clear cell carcinoma (KIRC)	531	223	17,662	16,531
Kidney renal papillary cell carcinoma (KIRP)	287	72	17,357	13,727
Acute myeloid leukemia (LAML)	151	92	16,477	14,445
Brain lower grade glioma (LGG)	511	201	17,801	14,036
Liver hepatocellular carcinoma (LIHC)	366	225	16,931	14,581
Lung adenocarcinoma (LUAD)	502	258	17,764	14,936
Lung squamous cell carcinoma (LUSC)	495	252	17,989	17,170
Mesothelioma (MESO)	85	80	17,562	16,705
Ovarian serous cystadenocarcinoma (OV)	302	231	17,968	16,849
Pancreatic adenocarcinoma (PAAD)	177	122	18,007	12,693
Pheochromocytoma and paraganglioma (PCPG)	179	23	17,373	14,346
Prostate adenocarcinoma (PRAD)	497	97	17,700	17,191
Rectum adenocarcinoma (READ)	94	29	17,575	15,504
Sarcoma (SARC)	259	153	17,375	15,299
Skin cutaneous melanoma (SKCM)	102	44	17,298	15,242
Stomach adenocarcinoma (STAD)	393	195	17,967	17,338
Testicular germ cell tumors (TGCT)	134	36	18,471	13,477
Thyroid carcinoma (THCA)	500	60	17,435	16,059
Thymoma (THYM)	119	24	17,646	17,055
Uterine corpus endometrial carcinoma (UCEC)	174	49	17,640	17,047
Uterine carcinosarcoma (UCS)	56	41	17,986	17,606
Uveal melanoma (UVM)	80	34	16,620	16,088

For each cancer type, both models were applied separately for every applicable gene. For the log-rank test, patients were divided into low and high gene expression groups based on the median expression value of each tested gene. We considered a gene as an SRG if it had a false discovery rate (FDR) less than 0.05. Furthermore, a gene could be interpreted as a harmful or a protective gene based on the restricted mean survival time (RMST), which is estimated from the area under the survival curve. That is, when the high-expression group had a lower RMST, it could be viewed as a harmful gene, and vice versa. Similarly, in the Cox regression test, genes with an FDR of less than 0.05 was regarded as an SRG. The positive and negative Cox coefficients were used to classify harmful and protective genes, respectively.

### Pan-cancer analysis of SRGs shows cancer specificity

**Fig. 2:**
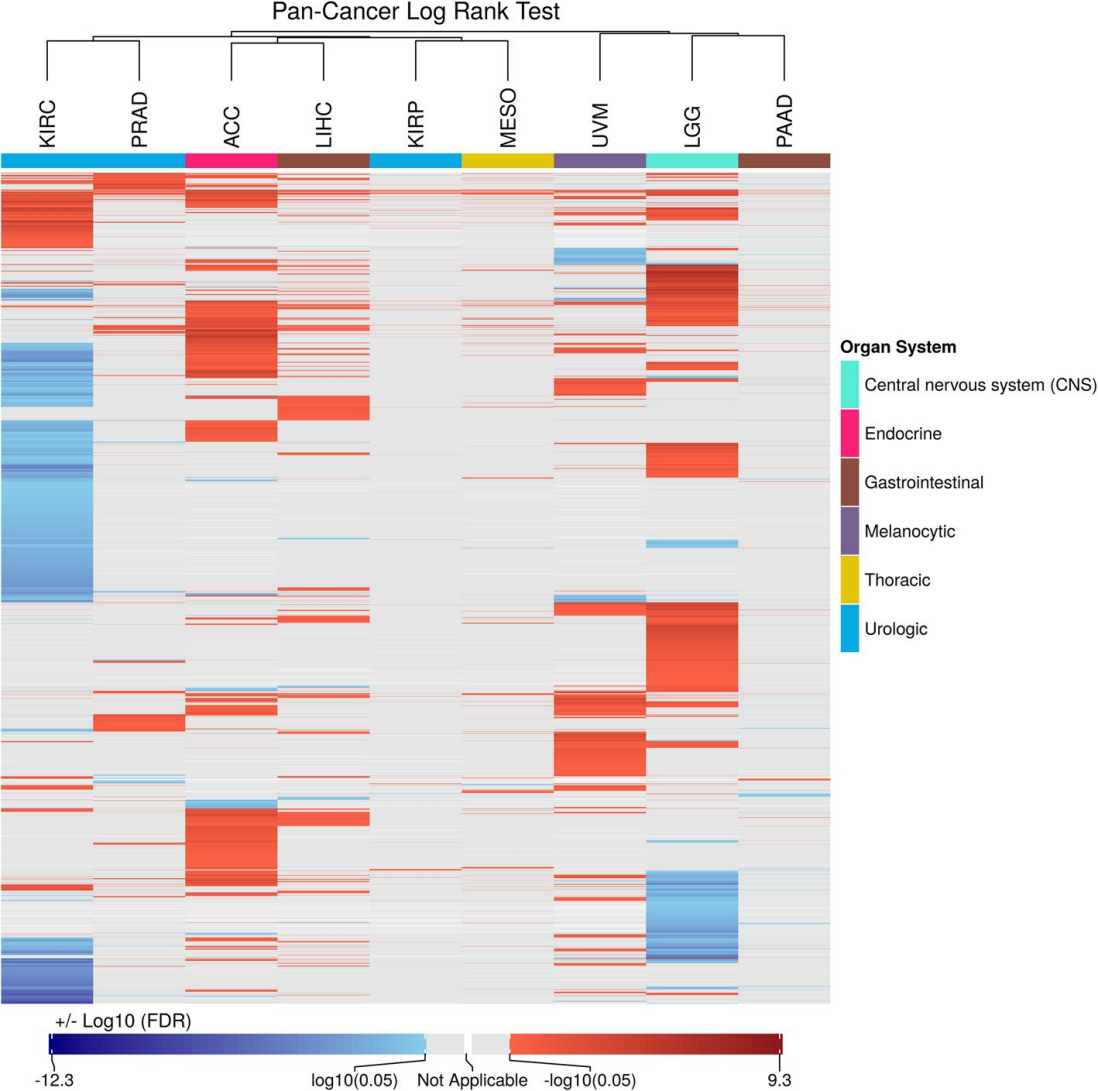
Pan-Cancer analysis of survival-related genes from the log-rank test. Benjamini & Hochberg adjusted *p*-values from the log-rank test were log10-transformed. Absolute values were taken for harmful genes and shown in red. Protective genes were shown in blue. Grey color indicates insignificant cases (FDR ≥ 0.05). White color indicates inapplicable cases. Each row represents the log p-values from a specific gene in cancer types. Each column represents a cancer type with the number of significant genes (FDR < 0.05) greater or equal to 100. Genes not significant in any cancer types were not shown here. Rows were clustering by Euclidean distance and complete linkage. Columns were clustered by Pearson distance and complete linkage. The organ system is indicated with different colors. The scale bar at the bottom indicates the range of log p-values

**Fig. 3:**
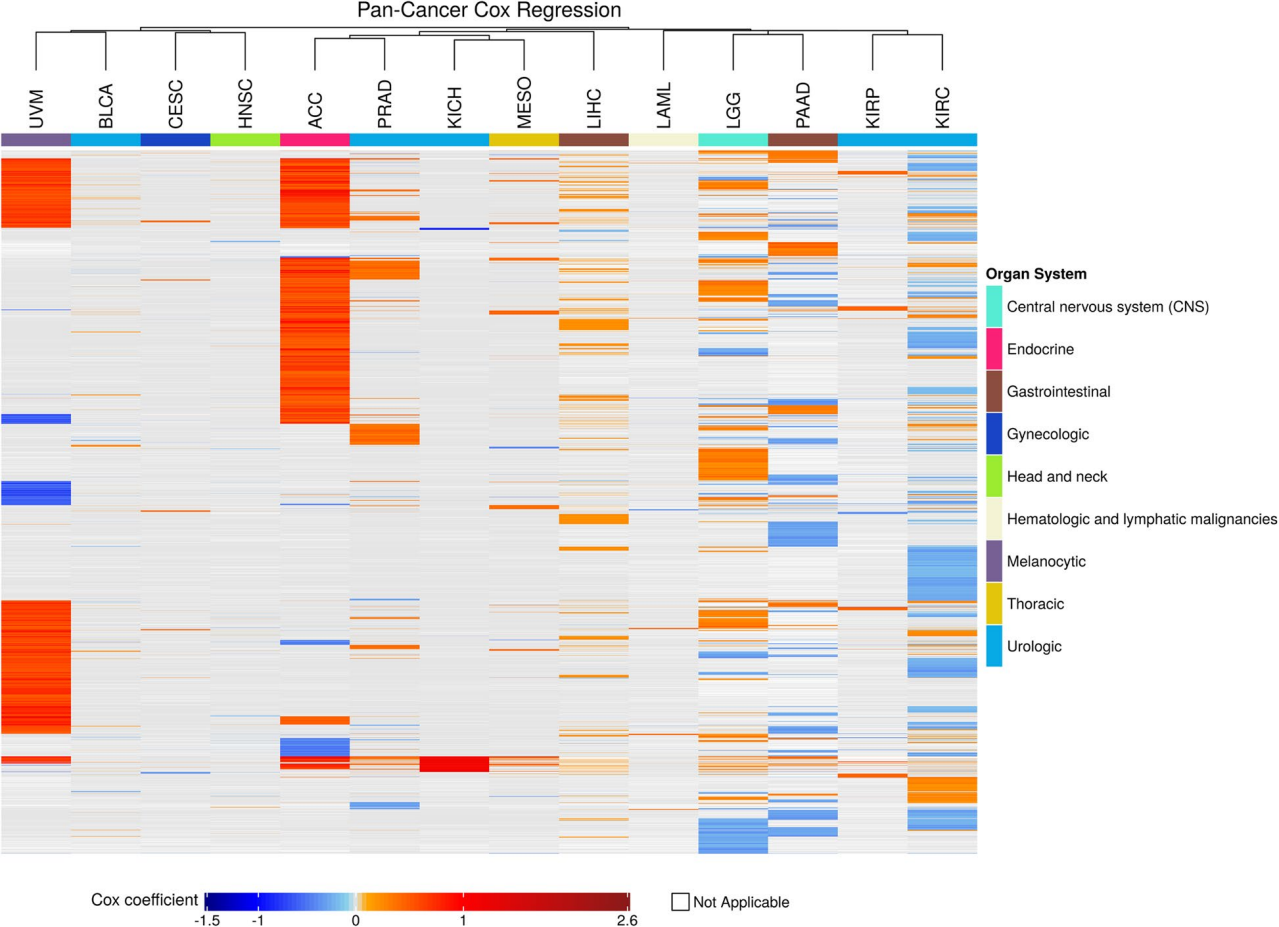
Pan-cancer analysis of survival-related genes from Cox regression. Significant regression coefficients from Cox regression were clustered. Harmful genes were shown in red and protective genes were shown in blue. Grey color indicates insignificant cases (FDR ≥ 0.05). White color indicates inapplicable cases. Each row represents the Cox coefficients from a specific gene in cancer types. Each column represents a cancer type with the number of significant genes (FDR < 0.05) greater or equal to 100. Genes not significant in any cancer types were not shown here. Rows and columns were clustering by Pearson distance and complete linkage. The organ system is indicated with different colors. The scale bar at the bottom indicates the range of Cox coefficients

We next investigated whether the SRGs were shared by different cancer types. We clustered *p*-values from the log-rank test and coefficient values from the Cox regression. For cancers having at least 100 SRGs from the log-rank test and Cox regression, the FDRs and coefficients were analyzed and plotted in Figs. 2 and 3, respectively. The heatmaps suggest that most of the cancer types have few or no SRGs, according to both the test and regression. In general, most of the SRGs were specific to certain cancer types, and the number of SRGs was highly diverse among cancer types. Still, for the SRGs identified by Cox regression (Fig. 3), squamous cell cancers (CESC and HNSC) were found to be clustered together. Notably, we observed that KICH was not grouped with the other two kidney-origin cancers (KIRC and KIRP). This result is consistent with previous clustering work based on the similarity of mRNA profiles [17]. Interestingly, we found that LGG and PAAD were clustered together, according to both the log-rank tests and Cox regressions.

Moreover, nine cancer types, ACC, KIRC, KIRP, LGG, LIHC, MESO, PAAD, PRAD, and UVM, had more than 100 SRGs discovered, under both models. The SRGs were moderately shared between the two models (Additional file 1: Supplementary Table 1). All the overlapping genes showed the same tendency to be harmful or protective, suggesting that the two models produced consistent results. Overall, Cox regression tended to identify more SRGs in more cancer types compared to the log-rank test, and most of the discovered SRGs were harmful, except for KIRC, with the majority of SRGs being protective.

### Cancer-related pathways were enriched with SRGs

**Fig. 4:**
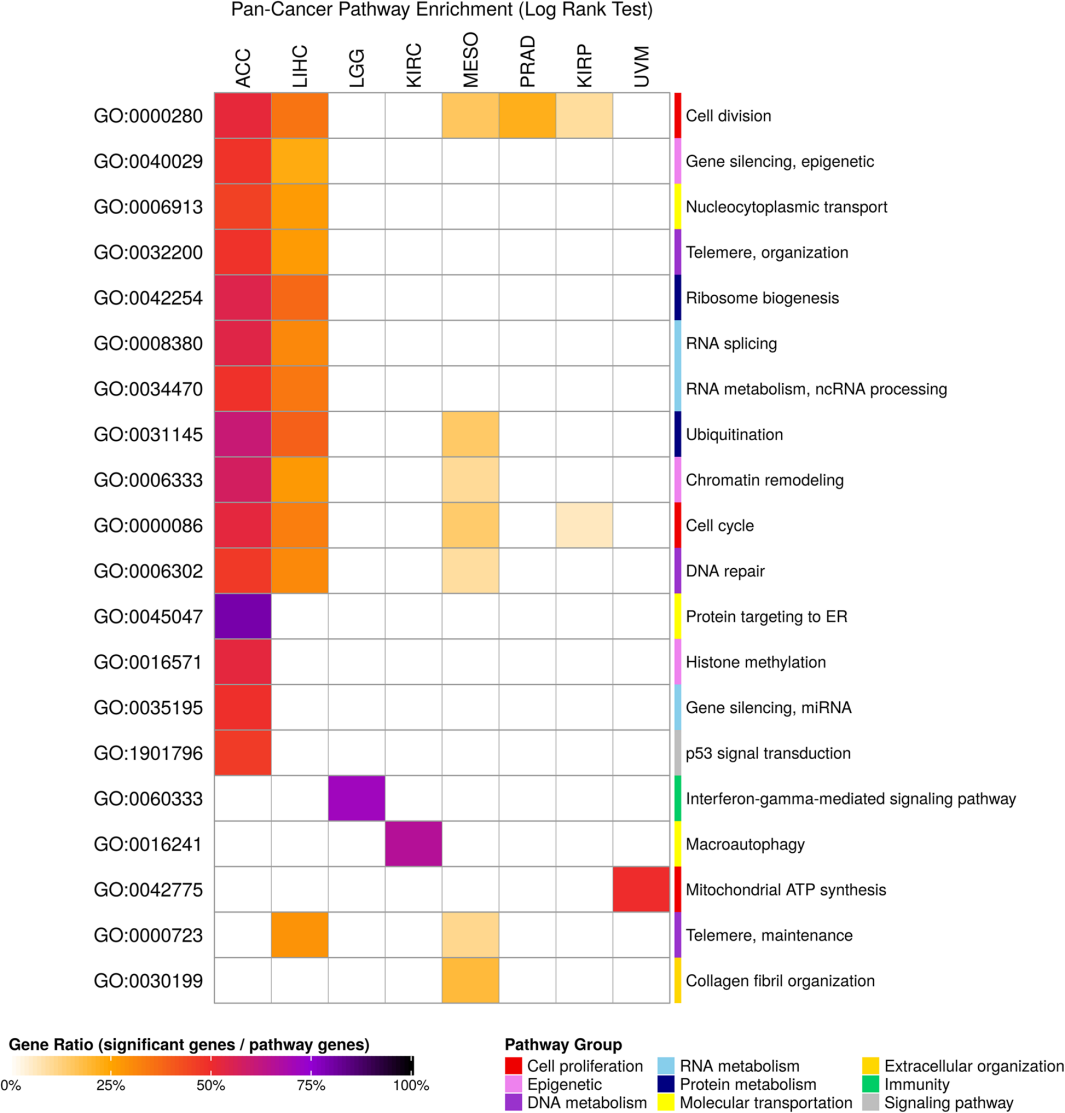
Pathway enrichment of survival-related genes from the log-rank test. SRGs from the log-rank test were enriched with Gene Ontology. Pathways having FDR < 0.001 are displayed in the heatmap. Significant pathways were manually grouped according to the relationship in Gene Ontology. The names of grouped pathways are shown on the right side and GO IDs that represented the grouped pathways are shown on the left side. The grouped pathways are further categorized by their biological functions as indicated in the bottom-right annotation legend. The gene ratio that indicates the percentage of significant genes (SRGs) enriched in the pathway is presented with different block colors

**Fig. 5:**
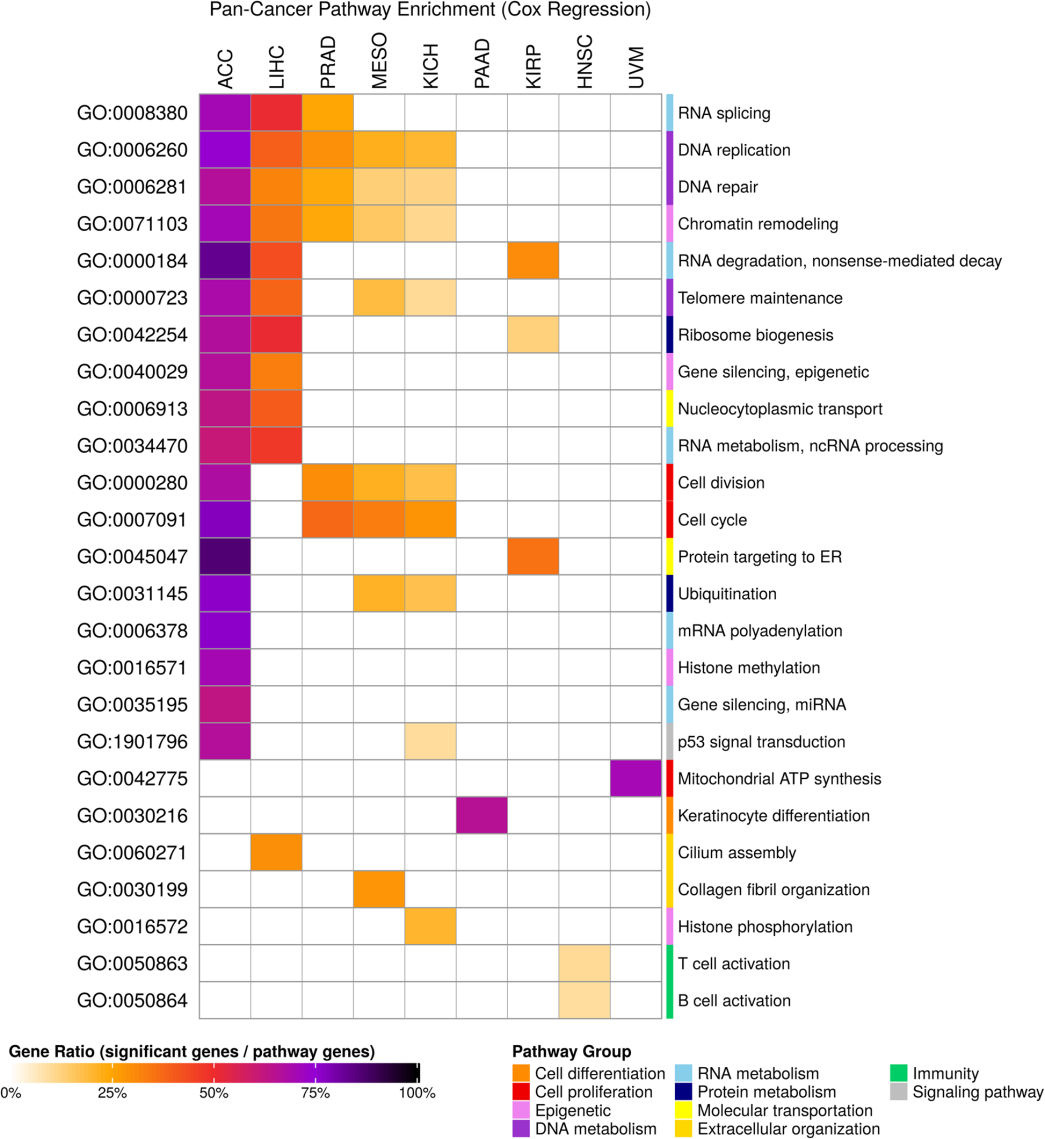
Pathway enrichment of survival-related genes from the Cox regression. SRGs from the Cox regression were enriched with Gene Ontology. Pathways having FDR < 0.001 are displayed in the heatmap. Significant pathways were manually grouped according to the relationship in Gene Ontology. The names of grouped pathways are shown on the right side and GO IDs that represented the grouped pathways are shown on the left side. The grouped pathways are further categorized by their biological functions as indicated in the bottom-right annotation legend. The gene ratio that indicates the percentage of significant genes (SRGs) enriched in the pathway is presented with different block colors

To date, several cancer hallmarks have been well studied and defined [25]. We were interested in whether the SRGs identified here may be involved in the hallmark pathways. We chose Gene Ontology (GO) terms for pathway enrichment analysis and organized similar pathways into one major pathway, such as cell cycle or DNA repair, and selected one GO term that could best represent the category. Cancers in both models had many hallmark-related pathways in common (Figs. 4 and 5). For example, cell division and cell cycle are the most frequent pathways shared among different cancer types. Enriched results from both models show that ACC and LIHC have the highest number of enriched pathways related to survival, and these pathways are concentrated in cell growth and molecular metabolism.

In addition, we noticed that cell division pathway in LIHC was discovered by the log-rank test, but not by Cox regression. Indeed, we found that the cell division pathway, GO:0000280, was enriched by Cox regression, but it had an FDR value larger than the FDR threshold, 0.001 (Additional file 2). This result suggests that with the multiple statistical correction steps, only the most significant and hence reliable pathways have been enriched in our analysis.

Finally, it is worth noting that LGG and KIRC have the highest number of SRGs according to the log-rank tests, but the SRGs seem to participate in diverse biological functions. Only about 1% of SRGs in LGG and KIRC are involved in enriched pathways (Additional file 3: Supplementary Table 2). This implies that the remaining SRGs may be scattered amongst distinct pathways, making over representation analysis in most pathways insignificant.

### SRGs are not over-represented by cancer driver genes

A cancer driver gene confers tumor cells a selective growth advantage over normal cells, and many driver genes have been found to be prognostic [26]. Hence, it may be intuitive to imagine that the SRGs would correlate with the driver genes. We examined whether the SRGs identified in this study are the same driver genes as in DriverDBV3 [27], an integrated driver gene database. All the mutation-based, CNV-based, and methylation-based driver genes were used in our analysis. We applied Fisher’s exact test and found that for most cancers the SRGs are mostly not significantly associated with the mutation-based and the methylation-based driver genes, and also, the SRGs identified using both the log-rank test and Cox regression are overrepresented with driver genes identified with CNV alterations in LGG and UVM (Table 2 and Additional file 4: Supplementary Table 3).

**Table 2:** Comparison between survival-related genes and cancer driver genes

Cancer Type	# of SRGs^a^	DriverDBV3					
		Mutation^b^		CNV		Methylation	
**Log-Rank Test**							
KIRC	7770	46.9%	(340/725) **	0%	(0/3)	50%	(6/12)
LGG	6691	32.5%	(570/1752)	82.2%	(37/45) ***	–	
ACC	5243	19.7%	(74/375)	32.3%	(40/124)	–	
UVM	3765	25%	(9/36)	62%	(75/121) ***	–	
LIHC	2359	9.5%	(106/1113)	9.6%	(8/83)	6.6%	(12/181)
PRAD	1538	5.1%	(58/1141)	23.5%	(4/17)	4.4%	(3/68)
MESO	586	2.5%	(1/40)	0%	(0/1)	–	
PAAD	443	1.6%	(16/971)	0%	(0/2)	16%	(4/25)**
KIRP	238	1.9%	(12/631)	1.1%	(1/87)	2.2%	(5/224)
BLCA	39	0.2%	(6/2404)	0%	(0/104)	0%	(0/443)
CESC	35	0.2%	(4/1815)	0%	(0/52)	–	
LAML	29	0%	(0/413)	0%	(0/3)	–	
HNSC	18	0%	(0/1914)	1%	(1/103)	0%	(0/89)
STAD	10	0.1%	(4/3695)	0%	(0/86)	–	
LUAD	8	0%	(1/2903)	0%	(0/37)	0%	(0/18)
SKCM	1	0%	(0/4559)	0%	(0/18)	–	
**Cox Regression**							
KIRC	7091	38.3%	(278/725)	0%	(0/3)	50%	(6/12)
ACC	6467	25.9%	(97/375)	43.5%	(54/124)	–	
UVM	5600	33.3%	(12/36)	84.3%	(102/121) ***	–	
LGG	5418	27.2%	(476/1752)	42.2%	(19/45) ***	–	
PAAD	4287	21.2%	(206/971)	0%	(0/2)	36%	(9/25)*
LIHC	2384	10%	(111/1113)	8.4%	(7/83)	9.9%	(18/181)
PRAD	2068	8.1%	(92/1141)	35.3%	(6/17) *	5.9%	(4/68)
MESO	728	2.5%	(1/40)	0%	(0/1)	–	
KIRP	592	2.4%	(15/631)	2.3%	(2/87)	1.8%	(4/224)
BLCA	442	2.7%	(64/2404)	1%	(1/104)	1.6%	(7/443)
KICH	407	3.7%	(2/54)	–		–	
CESC	230	1.4%	(25/1815)	0%	(0/52)	–	
HNSC	204	0.9%	(17/1914)	1.9%	(2/103)	0%	(0/89)
LAML	158	0.7%	(3/413)	0%	(0/3)	–	
LUAD	71	0.4%	(12/2903)	5.4%	(2/37) **	0%	(0/18)
PCPG	64	2%	(1/49)	0%	(0/5)	–	
BRCA	24	0.1%	(2/2162)	0%	(0/220)	0%	(0/36)
UCEC	5	0%	(0/6669)	0%	(0/76)	–	
STAD	3	0%	(1/3695)	0%	(0/86)	–	
SARC	2	0%	(0/543)	0%	(0/104)	–	
THCA	2	0%	(0/192)	0%	(0/3)	0%	(0/201)

Note: Fisher’s exact test of SRGs and cancer driver genes; *p < 0.05, **p < 0.01, ***p < 0.001, one-tailed. The first number in the parentheses indicates the count of overlapping genes between SRGs and cancer driver genes, and the second number indicates total driver genes that are also applicable genes in specified cancer

- Not available; no driver genes were described in those cancer types

^a^SRGs for both models are defined as FDR < 0.05

^b^Mutation-based driver genes were merged based on 14 tools summarized by DriverDBV3

## Discussion

In the present study, we applied two popular statistical tools, the log-rank test and the Cox proportional hazards model, on TCGA mRNA expression data, and revealed cancer-specific survival-related genes (SRGs). Although the log-rank test provides less information than Cox regression, it depends on fewer assumptions and so may be considered as having some advantages as regards robustness and power. It is partly for this reason that it is so commonly used in the literature, and why we chose to include it for our analysis alongside Cox regression. The two models identified different sets of SRGs across different cancer types, and the inconsistency may be due to the characteristics and limitations of these two models. For example, the log-rank test dichotomizes samples and further tests the null hypothesis of no difference between groups in the survival probability at any time point. As the name of the test suggests, it is a rank test and so ignores the quantitative trait, i.e., the values of the expression level. In contrast, Cox regression derives numerical estimates of coefficients whose scale is meaningful and quantifies the hazards of the genes. Although the rationales behind the two procedures are different, the common SRGs discovered under them share the same correlations between expression levels and the survival risk (Additional file 1: Supplementary Table 1). The SRGs identified in this work are based on the currently largest pan cancer dataset, TCGA and it will be worthwhile to validate these SRGs in other new large-scale cancer genomic datasets when they become available.

We utilized univariate Cox regression to discover SRGs. Other confounding factors such as gene-gene or gene-environment interactions were not considered and could potentially interfere with the statistical power of the model. For example, cancer subtype is one well-known factor that could lead to a different prognosis. To investigate whether cancer subtype affects the identified SRGs, we used BRCA as an example, because BRCA has a common classification system, PAM50 [28], based on its gene signature. When we stratified BRCA samples following PAM50 and fitted the Cox model, we found an increased number of SRGs in the LumA subtype (data not shown). This implies that cancer subtype can potentially affect model performance. Apart from this, some TCGA cancer types are a mixture of various tissues. Take HNSC for example, the data for which were collected from mucosal linings of the upper aerodigestive tract, encompassing oral cavity, nasal cavity, paranasal sinuses, pharynx and larynx [29]. The discrepancy between mRNA profiles and the diversity of tissue origins within the same cancer type may adversely affect the statistical power of SRGs. Hence, we subsequently examined the performance of our univariate regressions by calculating the concordance index (C-index), an indicator of the Cox model’s accuracy [30]. We found that the medians of the C-index were mostly around 0.6 (Additional file 5: Supplementary Fig. 1). In other studies [31–33], the C-index often ranged from 0.6 to 0.8 when multiple genes or clinical factors were included in the model variables. The C-indexes we calculated suggest that our method could be further improved by controlling for other variables using multivariate survival regression, such as the Cox-Lasso method [34, 35].

Another confounding factor may come from the transcriptional regulatory networks. Genes governed by the same transcription factor are potentially co-expressed. Therefore, they may be identified as SRGs together because our models discovered SRGs solely based on the correlation between mRNA expression levels and survival times. Ranking the importance of these SRGs in cancer survival would require further investigation and validation using other databases, such as cBioPortal [12]. Meanwhile, these important indexes can be used in multivariate survival analysis with filtered important genes which might provide better explanatory power [8, 36].

It is worth mentioning that the potentially dual characteristics of a gene in regulating cancer development have recently become more evident [37]. For example, Notch was found to be both tumor suppressive and oncogenic in HNSC [38]. Studies have discovered that many cancer driver genes may have an opposite effect among different cancer types [38, 39]. Our study provides an avenue to explore such dual characteristic genes based on our clustering results. We found that some genes are harmful in one cancer whilst being protective in others (Figs. 2 and 3). The functions of these genes and underlying mechanisms related to survival are worthy of further investigation.

Interestingly, some pathways enriched with SRGs have been found to be dominant in specific cancer types. For example, in a recent study [40], survival-related pathway in mitochondrial ATP synthesis was enriched from both models in uveal melanoma (UVM), a common primary intraocular tumor in adults. An in vitro study demonstrated that knockdown of histone subunit macroH2A1 leads to dysregulation of mitochondrial metabolism and is related to UVM aggressiveness. Another study reported that ATP synthase transporters were upregulated in a uveal melanoma cell line [41]. Also, autophagy pathways enriched by SRGs from the log-rank test of kidney renal clear cell carcinoma (KIRC) were evidenced by recent studies as potential therapeutic targets [42, 43]. Keratinocyte differentiation pathways enriched by SRGs from Cox regression of pancreatic adenocarcinoma (PAAD) were correlated in cancer progression and invasion [44, 45]. Together, these findings are consistent with the survival-related pathways found in our study to be biologically significant.

Moreover, we found that SRGs are not over-represented by known cancer driver genes, given that the driver genes we tested were derived from mutations, CNV and methylation, even though we found that the SRGs in LGG and UVM were both enriched in CNV-based, but not mutation-based or methylation-based driver genes. We reasoned that the difference may be due to the distinctive biological outcomes of CNV and mutation. Recently, CNVs were found to be directly correlated with mRNA expression, and it was deduced that the mutation of driver genes may result in protein malfunction but not necessarily induce mRNA expression level changes [46]. Another reasonable explanation is that these survival related genes are the consequence of tumor growth. In one study, energy metabolism was altered to compensate the unusually rapid proliferation rate in tumor cells [47]. Thus, the high glucose uptake rate may lead to gene expression changes in glycolysis pathway. Our pathway enrichment results using the log-rank test demonstrated that KIRC has a pathway, named acetyl-CoA biosynthetic process (GO:0006085, Additional file 2, sheet 2_pathway_logrank test), with FDR less than 0.05. This pathway was previously reported to be associated with tumorigenesis in KIRC [48]. Collectively, although most of the SRGs in the two models were not correlated with cancer driver genes, they may be part of other factors in cancer driver pathways.

In large-scale biomedical research, one should always be cautious of multiple statistical tests and make appropriate adjustments. However, the cost of such corrections is a loss of statistical power for detecting true positives. In pathway enrichment analysis, clusterProfiler [49] generally tests thousands of pathways for each query, and is subsequently corrected by the Benjamini & Hochberg method to control the false discovery rate. Such a high number of tests could result in some pathways being found to be insignificant even if they are biologically significant. Given that GO terms are organized in a directed acyclic graph, many of them are highly correlated and can be clustered into groups. It is possible to condense the pathways from thousands to hundreds but still provide biologically representative clusters. Accordingly, to maximize the statistical power, we could focus on specific pathways or remove superfluous pathways to reduce the number of statistical tests. Such a strategy may help to unveil novel survival-related pathways in the future.

Finally, we would like to make four caveats regarding the research. Firstly, the focus is on associations between genes and survival time, and the predictive power of genes on survival time is not examined. Secondly, genes are considered for significance of association only singly, and no gene-to-gene or gene-to-environment interactions are considered. Incorporating potentially confounding demographic, clinical and other covariates into the analysis would likely improve statistical power as regards cancer prognosis. Thirdly, reproducibility of the results in the research is not examined, due to limitations of available data. And fourthly, in the analysis based on the log-rank test, gene expression is dichotomized into high-expression and low-expression groups, whereby important information regarding quantitative traits of gene expression is likely lost. This may partly account for the inconsistency between our results based on the log-rank test and those based on Cox regression. These caveats suggest the need for further research.

## Conclusions

This work provides a comprehensive analysis of the SRGs in cancers based on data in TCGA. We discovered that the SRGs in different cancer types are significantly involved in cancer hallmark pathways; however, they vary widely in number. We also found that the SRGs are not over-represented by cancer driver genes. These findings are supported by statistical analyses using the log-rank test and Cox regression. In summary, our pan-cancer analysis reveals the distributions and biological functions of SRGs in 33 cancer types and provides potentially valuable clinical insights.

## Methods

### Data processing from TCGA

TCGA mRNA expression and clinical data [1] were downloaded from Broad GDAC Firehose [50] through firehose_get (version 0.4.13) with keyword “Level_3__RSEM_genes__data” and “Merge_Clinical.Level_1”, respectively. The data versions are both “stddata__2016_01_28”. For mRNA expression data, filenames contain “illuminahiseq_rnaseqv2” were used. Primary solid tumor (sample type code 01) and primary blood derived cancer (sample type code 03) were selected for downstream analysis. As described in TCGA publication [51], the sequencing raw reads were aligned to hg19 genome by MapSplice, translated the genome coordinates to the transcriptome based on UCSC knownGene, and quantified the transcriptome with RSEM. The resulting values (shown in “scaled_estimate” column from the downloaded expression matrix), which is the estimated frequency of a transcript among total transcripts, were multiplied by 10^6^ to obtain transcript per million (TPM) and used throughout this study as gene expression values. For clinical data, survival time were parsed from three attributes: days_to_death as overall-survival (OS) time, days_to_last_followup as follow-up time and days_to_new_tumor_event_after_initial_treatment as disease-free-survival (DFS) time. The study used DFS time predominantly and used OS time instead if DFS time did not exist.

### Log-rank test

The log-rank test was conducted individually for each gene in every cancer type. Gene expression values were divided into two groups, the high-expression group and the low-expression group, based on the median value. If the median value was equal to zero, we removed that gene from the test. To determine the impact of gene expression on survival, we compared the restricted mean survival time (RMST) between two groups, where higher RMST means better survival. Benjamini & Hochberg multiple test correction [52] were applied to the resulting *p*-values for each cancer type.

### Cox proportional hazards model

An “event” was considered to occur if a patient died or relapsed before the end of the study. Otherwise, the patient was considered as censored, for example, if they were still alive or cancer-free healthy at the end of the study, or if they could not be contacted at that time. That is, if either of OS and DFS time existed, a patient was considered having an event; Otherwise, a patient was defined as censored. Before fitting the Cox model, genes in each cancer were screened separately to meet the following criterions: (1) MAD > 0, and (2) proportional hazards assumption. For MAD, it was calculated for each gene in every cancer, defined by the following equation:

$${MAD}_j= median\ \left(|{X}_{ij}-\overset{\sim }{X_j}|\right)$$

, where *MAD*_*j*_ is the median absolute deviation of gene *j*, *X*_*ij*_ is the expression value of gene *j* in sample *i*, and $\overset{\sim }{X_j}$ is the median expression value of gene *j*. To test the assumption of proportional hazards for each gene, we obtained Schoenfeld residuals [53] for each gene and tested the null hypothesis that the correlation between the Schoenfeld residuals and ranked failure time were zero by using the function cox.zph in R package. Genes with *p*-value of less than 0.05 were considered to be violating the assumption of the test and were excluded from fitting the Cox model. Genes passed above thresholds were log2 transformed as equation:

$${\mathit{\log}}_2\left( Gene\ value+{10}^{-5}\right)$$

, where a small value 10^−5^ was added to prevent zeros when taking logarithms. The transformed values were further standardized to approach ~N (0, 1) normal distribution. We started to apply the model on each gene of every cancer with following hazard function:

$${h}_{ij}(t)={h}_{0_{ij}}\ (t)\ {\mathit{\exp}}^{\beta_{ij}\times {X}_{ij}}$$

, where *i* indicates gene, *j* indicates cancer, *h*_*ij*_(*t*) is the hazard of gene *i* in cancer *j* at time *t*, ${h}_{0_{ij}}$ is the baseline hazard, *β*_*ij*_ is the Cox coefficient, and *X*_*ij*_ is the transformed and standardized gene values. The resulting Wald *p*-value for Cox coefficients were corrected with Benjamini & Hochberg method [52] for each cancer.

The concordance index (C-index) evaluates the accuracy of the Cox model [30]. The C-index is interpreted similarly to the AUC (area under the receiver operating characteristics curve). A C-index of 1 means that the SRGs are perfect at discriminating which patient have a better survival, while a C-index of 0.5 indicates the survival prediction of the gene is random.

### Heatmap clustering

For the log-rank test, we used the Benjamini & Hochberg method of adjusted p-value (also known as FDR) to produce the heatmap. Before clustering, FDR values were log10 transformed, and genes whose RMST value was lower on the high expression group were multiplied by − 1 to become positive log FDR. The distances of column and row values were calculated by Pearson correlation and Euclidean distance, respectively. Both columns and rows used complete-linkage to draw the column dendrogram and the order of rows. For Cox regression, we used Cox coefficients to generate the heatmap. Genes with cox coefficients with FDR < 0.05 were preserved and those with FDR ≥ 0.05 were changed to zero. The distances of column and row values were both calculated by Pearson correlation and ordered by complete-linkage. The organ system annotations in the heatmap were classified according to a previous study [54]. All heatmaps in the papers were generated by R package ComplexHeatmap [55].

### Pathway enrichment analysis

Pathway analysis was performed by R package clusterProfiler [49]. Cancer-specific SRGs (FDR < 0.05) from the log-rank test and Cox regression were selected for Gene Ontology enrichment [56, 57]. Function dropGo was run to remove level 1 to level 5 GO terms, which may contain general but limited information about pathways. The remaining pathways with FDR less than 0.001 were manually grouped according to the pathway relationships in the directed acyclic graph. To give an overall picture of the enriched pathway, we picked a GO term that was significant in most cancer types for each manually separated group. For each represented GO term, we showed the ratio of significant genes (SRGs) enriched in a pathway to all genes comprising the pathway. Of note, because applicable genes in each cancer type were different, the numbers of genes involved in the pathway may have subtle difference. Detailed pathway enrichment and grouped results could be found in Additional file 2.

### Statistical test for driver genes association

One-tailed Fisher’s exact test was performed to test the linkage between SRGs and cancer driver genes from DriverDBV3 [27]. We downloaded three types of driver genes, including the mutation-based, the CNV-based, and the methylation-based from DriverDBV3 database (http://driverdb.tms.cmu.edu.tw/download). The downloaded tables described cancer-specific driver genes. Specifically, the mutation-based drivers were categorized by 14 different tools. We merged all mutation-based driver genes from the 14 tools. Fisher’s exact test was performed separately for each type of cancer and each type of driver gene as shown in the following table:

**Table:** 

	SRGs	Non-SRGs	
Driver genes	a	b	R1
Not driver genes	c	d	R2
	C1	C2	Applicable genes

The number of applicable genes depends on each cancer type in each model. C1 and C2 are the numbers of SRGs and non-SRGs in survival models, respectively. R1 and R2 are the numbers of driver genes and non-driver genes, respectively. All the driver genes not analyzed or not applicable in survival models were excluded in this analysis. Symbol *a* represents the number of SRGs that are also noted as driver genes in DriverDBV3. The number of *b*, *c* and *d* are derived accordingly.

## Supplementary Information

**Additional file 1: Supplementary Table 1.** Comparison of survival-related genes identified in the log-rank test and the Cox regression.

**Additional file 2.** Pathway enrichment results for survival-related genes. Enriched pathways for survival-related genes identified in different cancer types were listed in the excel file.

**Additional file 3: Supplementary Table 2.** Percentage of survival-related genes participated in enriched pathways.

**Additional file 4: Supplementary Table 3.** *p* values from Fisher exact test for comparison between survival-related genes and cancer driver genes.

**Additional file 5: Supplementary Figure 1.** Concordance index of survival-related genes. The concordance indexes for cancer with at least 100 SRGs are summarized in boxplot.

## Data Availability

All the transcriptome data and clinical information for cancers were downloaded from the TCGA database via Broad GDAC Firehose [1, 50]. Driver genes were downloaded from DriverDBV3 [27].

## Abbreviations

AUC: Area under the receiver operating characteristics curve
C-index: Concordance index
CNV: Copy number variation
DFS: Disease-free survival
FDR: False discovery rate
GO: Gene Ontology
MAD: Median absolute deviation
NGS: Next Generation Sequencing
OS: Overall survival
RMST: Restricted mean survival time
SRG: Survival-related gene
TCGA: The Cancer Genome Atlas
TPM: Transcript per million
