# Supplementary material for: Survival-related genes are diversified across cancers but generally enriched in cancer hallmark pathways
Source: BMC Genomics. 2022 May 4;22(Suppl 5):918. doi: 10.1186/s12864-022-08581-x (PMC9066720; doi:10.1186/s12864-022-08581-x)
Supplement: Supplementary file 1 — Additional file 1: Supplementary Table 1. Comparison of survival-related genes identified in the log-rank test and the Cox regression. [file 12864_2022_8581_MOESM1_ESM.docx]

**Supplementary Table 1.** Comparison of survival-related genes identified in the log-rank test and Cox regression

| Cancer Types | # of SRGs | | # of Common SRGs^†^ | Overlapping % ^§^ | Prediction Similarity % ^⁋^ |
| --- | --- | --- | --- | --- | --- |
|  | Log-Rank Test | Cox Regression |  |  |  |
| KIRC | 7770 | 7091 | 5842 | 68% | 100% |
| ACC | 5243 | 6467 | 4475 | 65% | 100% |
| LGG | 6691 | 5418 | 3777 | 64% | 100% |
| UVM | 3765 | 5600 | 3181 | 52% | 100% |
| LIHC | 2359 | 2384 | 1179 | 43% | 100% |
| PRAD | 1538 | 2068 | 1152 | 48% | 100% |
| PAAD | 443 | 4287 | 382 | 9% | 100% |
| MESO | 586 | 728 | 368 | 40% | 100% |
| KIRP | 238 | 592 | 67 | 9% | 100% |
| BLCA | 39 | 442 | 24 | 5% | 100% |
| CESC | 35 | 230 | 21 | 9% | 100% |
| LAML | 29 | 158 | 18 | 11% | 100% |
| HNSC | 18 | 204 | 12 | 6% | 100% |
| LUAD | 8 | 71 | 2 | 3% | 100% |
| BRCA | 0 | 24 | 0 | - | - |
| KICH | 0 | 407 | 0 | - | - |
| PCPG | 0 | 64 | 0 | - | - |
| SARC | 0 | 2 | 0 | - | - |
| SKCM | 1 | 0 | 0 | - | - |
| STAD | 10 | 3 | 0 | - | - |
| THCA | 0 | 2 | 0 | - | - |
| UCEC | 0 | 5 | 0 | - | - |

† Common SRGs were matched by EntrezID.

§ Overlapping percentages were calculated by the number of common SRGs divided by the number of genes that are SRGs in one of the models and are applicable in both models.

⁋ Prediction similarity indicated whether SRGs had the same positive or negative correlation with survival outcome.

- Not available.
