# Supplementary material for: Survival-related genes are diversified across cancers but generally enriched in cancer hallmark pathways
Source: BMC Genomics. 2022 May 4;22(Suppl 5):918. doi: 10.1186/s12864-022-08581-x (PMC9066720; doi:10.1186/s12864-022-08581-x)
Supplement: Supplementary file 3 — Additional file 3: Supplementary Table 2. Percentage of survival-related genes participated in enriched pathways. [file 12864_2022_8581_MOESM3_ESM.docx]

**Supplementary Table 2.** Percentage of survival-related genes participated in enriched pathways

| Cancer Type | Log-Rank Test^†^ | Cox Regression^†^ |
| --- | --- | --- |
| ACC | 33.4% | 35.7% |
| CESC | - | 3.0% |
| HNSC | - | 19.6% |
| KICH | - | 38.3% |
| KIRC | 1.4% | - |
| KIRP | 20.2% | 18.4% |
| LGG | 0.9% | - |
| LIHC | 34.1% | 35.2% |
| MESO | 29.7% | 29.7% |
| PAAD | - | 1.8% |
| PRAD | 9.9% | 17.5% |
| UVM | 1.1% | 1.0% |

† Percentage was defined as the number of SRGs enriched in all pathways (FDR < 0.001) over total SRGs.
