# Supplementary material for: Survival-related genes are diversified across cancers but generally enriched in cancer hallmark pathways
Source: BMC Genomics. 2022 May 4;22(Suppl 5):918. doi: 10.1186/s12864-022-08581-x (PMC9066720; doi:10.1186/s12864-022-08581-x)
Supplement: Supplementary file 4 — Additional file 4: Supplementary Table 3. p values from Fisher exact test for comparison between survival-related genes and cancer driver genes. [file 12864_2022_8581_MOESM4_ESM.docx]

## Supplementary Table 3 – p values from Fisher exact test for comparison between survival-related genes and cancer driver genes

| Cancer Type | DriverDBV3 | | |
| --- | --- | --- | --- |
|  | Mutation | CNV | Methylation |
| \| Log-rank test \|  \|  \|  \| \| --- \| --- \| --- \| --- \| \| KIRC \| 0.005915 \| 1 \| 0.444584 \| \| LGG \| 0.811151 \| 7.14E-11 \| 1 \| \| ACC \| 0.999539 \| 0.297526 \| 1 \| \| UVM \| 0.395312 \| 7.58E-21 \| 1 \| \| LIHC \| 0.999075 \| 0.892931 \| 0.999263 \| \| PRAD \| 0.999957 \| 0.054121 \| 0.942231 \| \| MESO \| 0.734196 \| 1 \| 1 \| \| PAAD \| 0.929796 \| 1 \| 0.003034 \| \| KIRP \| 0.12908 \| 0.687323 \| 0.189993 \| \| BLCA \| 0.383479 \| 1 \| 1 \| \| CESC \| 0.432234 \| 1 \| 1 \| \| LAML \| 1 \| 1 \| 1 \| \| HNSC \| 1 \| 0.097929 \| 1 \| \| STAD \| 0.104618 \| 1 \| 1 \| \| LUAD \| 0.710138 \| 1 \| 1 \| \| SKCM \| 1 \| 1 \| 1 \| | | | |

| Cox Regression |  |  |  |
| --- | --- | --- | --- |
| KIRC | 0.431099971 | 1 | 0.413763673 |
| ACC | 0.999677562 | 0.091036254 | 1 |
| UVM | 0.490615402 | 2.90243E-31 | 1 |
| LGG | 0.737264594 | 1.73806E-07 | 1 |
| PAAD | 0.154024902 | 1 | 0.033493406 |
| LIHC | 0.998093682 | 0.937242429 | 0.964187927 |
| PRAD | 0.99879444 | 0.011472458 | 0.964822657 |
| MESO | 0.80803864 | 1 | 1 |
| KIRP | 0.899535678 | 0.641532623 | 0.91643981 |
| BLCA | 0.162050857 | 0.922881411 | 0.898119496 |
| KICH | 0.343664243 | 1 | 1 |
| CESC | 0.278550963 | 1 | 1 |
| HNSC | 0.810776992 | 0.2658686 | 1 |
| LAML | 0.629644794 | 1 | 1 |
| LUAD | 0.310138203 | 0.007775385 | 1 |
| PCPG | 0.171433316 | 1 | 1 |
| BRCA | 0.721008949 | 1 | 1 |
| UCEC | 1 | 1 | 1 |
| STAD | 0.468442055 | 1 | 1 |
| SARC | 1 | 1 | 1 |
| THCA | 1 | 1 | 1 |
