# Supplementary figures and images for: Survival-related genes are diversified across cancers but generally enriched in cancer hallmark pathways
Source: BMC Genomics. 2022 May 4;22(Suppl 5):918. doi: 10.1186/s12864-022-08581-x (PMC9066720; doi:10.1186/s12864-022-08581-x)

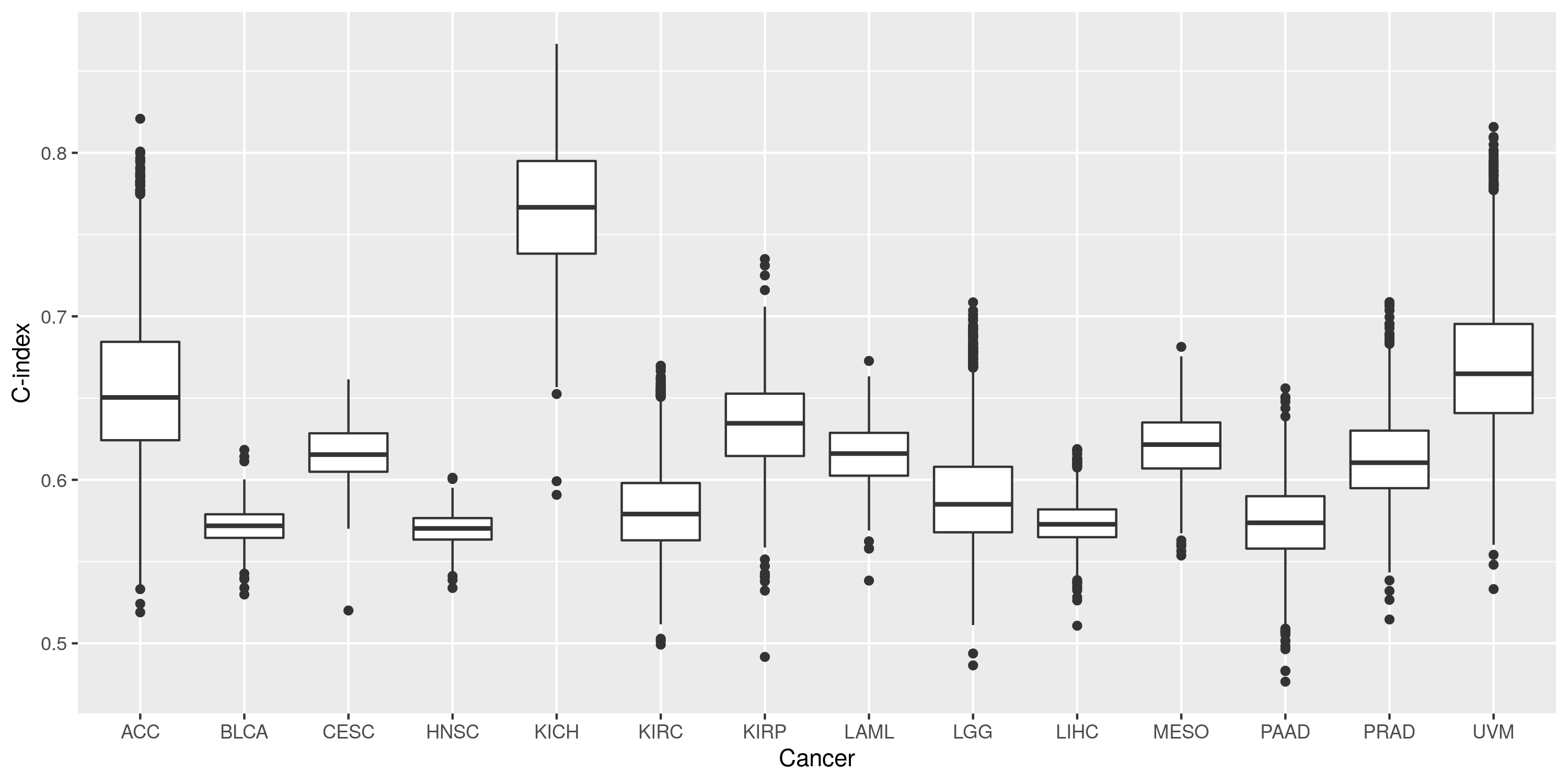

Supplement: Supplementary file 5 — Additional file 5: Supplementary Figure 1. Concordance index of survival-related genes. The concordance indexes for cancer with at least 100 SRGs are summarized in boxplot. [file 12864_2022_8581_MOESM5_ESM.png]
